# Feasibility of Fecal MicroRNAs as Novel Biomarkers for Pancreatic Cancer

**DOI:** 10.1371/journal.pone.0042933

**Published:** 2012-08-08

**Authors:** Alexander Link, Verena Becker, Ajay Goel, Thomas Wex, Peter Malfertheiner

**Affiliations:** 1 Department of Gastroenterology, Hepatology and Infectious Diseases, Otto-von-Guericke University, Magdeburg, Germany; 2 Gastrointestinal Cancer Research Laboratory, Division of Gastroenterology, Baylor Research Institute, Baylor University Medical Center, Dallas, Texas, United States of America; Deutsches Krebsforschungszentrum, Germany

## Abstract

**Introduction:**

Pancreatic cancer (PCA) is an aggressive tumor that associates with high mortality rates. Majority of PCA patients are diagnosed usually at late tumor stages when the therapeutic options are limited. MicroRNAs (miRNA) are involved in tumor development and are commonly dysregulated in PCA. As a proof-of-principle study, we aimed to evaluate the potential of fecal miRNAs as biomarkers for pancreatic cancer.

**Materials and Methods:**

Total RNA was extracted from feces using Qiagen's miRNA Mini Kit. For miRNA expression analyses we selected a subset of 7 miRNAs that are frequently dysregulated in PCA (miR-21, -143, -155, -196a, -210, -216a, -375). Subsequently, expression levels of these miRNAs were determined in fecal samples from controls (n = 15), chronic pancreatitis (n = 15) and PCA patients (n = 15) using quantitative TaqMan-PCR assays.

**Results:**

All selected miRNAs were detectable in fecal samples with high reproducibility. Four of seven miRNAs (miR-216a, -196a, -143 und -155) were detected at lower concentrations in feces of PCA patients when compared to controls (p<0.05). Analysis of fecal miRNA expression in controls and patients with chronic pancreatitis and PCA revealed that the expression of miR-216a, -196a, -143 und -155 were highest in controls and lowest in PCA. The expression of the remaining three miRNAs (miR-21, -210 and -375) remained unchanged among controls and the patients with either chronic pancreatitis or PCA.

**Conclusion:**

Our data provide novel evidence for the differential expression of miRNAs in feces of patients with PCA. If successfully validated in large-scale prospective studies, the fecal miRNA biomarkers may offer novel tools for PCA screening research.

## Introduction

Pancreatic cancer (PCA) is the second leading cause of gastrointestinal cancer-related deaths in Europe and USA [1], [2]. Despite intensive basic and clinical research the median survival of patients with PCA remains approximately 6–10 months after diagnosis, which highlights the aggressive tumor biology of this disease [3]. The majority of patients with PCA (>80%) present with regional or distant tumor dissemination at diagnosis that renders them ineligible for surgical tumor removal, which is at present the only potential curative therapy for this malignancy [1], [3]. Besides potential cancer-preventive lifestyle modifications (diet, physical exercise, cessation of smoking etc.), screening and detection of early PCA is considered as most promising strategy for reducing PCA-associated mortality [4]–[6]. Current clinical screening and diagnostic management of PCA patients is based on imaging techniques such as endoluminal ultrasound (EUS) or contrast-enhanced multi-detector CT scan. However, due to their invasive nature, use of radiation, and low sensitivity/specificity, such techniques at best may only be used for the screening in high-risk patients (e.g. hereditary PCA, Peutz-Jeghers syndrome etc.) [6], [7]. Therefore, there is an immediate need for identification and development of novel biomarkers, preferably non-invasive diagnostic technologies that could predict the development of PCA or its diagnosis at earliest stages.

The importance of such non-invasive biomarkers has long been recognized. Consequently, blood, stool or other body fluids are currently believed to be the best avenues for biomarker research that incorporates a broad spectrum of genetic and/or epigenetic alterations associated with cancer [6], [8]. Based upon this paradigm, multiple biomarkers such as hTERT, CA72-4, Osteopontin, REG4, MIC-1, *K-ras*, *p-53* or aberrant methylation of the CpG islands of the tumor suppressor genes are currently under evaluation [3], [9]–[11]. Nevertheless, till date, only carbohydrate antigen 19-9 (CA19-9) has found its clinical usefulness in the early detection of recurrent disease, following surgical treatment of PCA patients [12]. However, the use of CA19-9 for the PCA-screening is not recommended due to low sensitivity and specificity [13].

MicroRNAs (miRNAs), the small non-coding transcripts of ∼22 nucleotides, have recently been identified as a new class of cellular molecules with significant diagnostic, prognostic and therapeutic implications [14]. MiRNAs play an important role in a wide range of physiological and pathological processes [15]. By virtue of regulating gene expression, miRNAs are involved at the earliest steps in cancer pathogenesis of multiple human cancers [15], [16]. In addition, existence of unique miRNA expression patterns permit identification of different types and subtypes of cancers [17]–[19]. One of the most exciting features of miRNAs is their biological stability compared to DNA or mRNA. MiRNAs are remarkably protected from endogenous and exogenous degradation due to their small size and exosomal presence in body fluids [20], [21]. Based on this paradigm, several research groups have independently shown the potential of miRNA as blood-based biomarkers for PCA [21]–[23]. While blood-based approach is still a matter of the intensive research, it has also been suggested that gastrointestinal cancer-related genetic and epigenetic alterations may potentially be detected in feces [8], [24]. Considering the high volume (∼1.5 l) of pancreatic juice production and excretion into the bowel, it has been hypothesized that precancerous or early cancer-related molecular changes may be detectable in feces and blood of pancreatic cancer patients, providing a strong rationale for fecal biomarker research [8].

Taking into account that PCA is associated with unique alteration pattern of miRNA expression, we hypothesized that miRNA expression signature may be identifiable in feces of patients with pancreatic neoplasia. Accordingly, in the present study, we evaluated the potential of fecal miRNA expression analyses for identifying PCA. As a proof of principal, we demonstrate that several fecal miRNAs are differentially expressed in patients with pancreatic disease. Pilot analyses of stool specimens from patients with chronic pancreatitis and PCA suggest a potential role for fecal miRNAs as novel biomarkers in the early detection of PCA.

## Materials and Methods

### Clinical samples

The study was performed on the archived fecal specimens from consecutive patients that have been collected for clinical analyses and prospectively collected feces from healthy subjects. All prospectively recruited healthy volunteers provided written informed consent and the protocol was approved by the Institutional Review Board at Baylor University Medical Center [24]. The archived fecal samples were de-identified, since the written informed consent could not be obtained due to patient's migration or death, and the protocol was approved by the Institutional Review Board of Otto-von-Guericke University Magdeburg (Magdeburg, Germany). A total of 45 stool specimens, including 15 controls, 15 patients with chronic pancreatitis and 15 PCA patients were included in the study. Control samples were randomly selected from the stool samples collected for detection of *Helicobacter pylori* in feces from patients with non-malignant GI-disorders such as reflux or gastritis. Samples from patients with chronic pancreatitis and PCA were selected from consecutive patients with available frozen stool samples, which were collected for analyzing fecal *elastase* levels as a routine diagnostic test for exocrine pancreas insufficiency. Clinical and demographical data of the patients are presented in **Table 1** **.**

**Table 1:** Clinico-pathological characteristics of patients included to the study.

	Total	Controls	Chronic pancreatitis	Pancreatic cancer
	n (%)	n (%)	n (%)	n (%)
**Number**	45 (100%)	15 (33.3%)	15 (33.3%)	15 (33.3%)
**Sex**				
**Women**	24	10	2	12
**Men**	21	5	13	3
**Age (mean ± SD)**	55.1±16.6	38.5±9.8	55.0±9.5	71.7±9.7
**Diagnosis**				
**Gastritis**		11		
**Reflux**		4		
**Chronic pancreatitis**			15	
**Pancreatic tumor**				15
**UICC-Stage** *				
**IIB**				3
**III**				1
**IV**				11
**Localization**				
**Head**				11
**Corpus**				2
**Tail**				2
**Ca 19-9 (U/ml) (mean±SD)**				20140±24892
**Endocrine pancreatic insufficiency** #	18		8	10
**Exocrine pancreatic insufficiency** §	25		12	13
**Prior pancreatic surgery** *	3		3	

* *- at time point of sample collection;*

# *- endocrine pancreatic insufficiency is defined by impaired glucose tolerance test or by manifest diabetes mellitus;*

§ - exocrine pancreatic insufficiency is defined by reduced elastase in stool.

### Stool samples collection

All samples were collected and processed uniformly by using feces sample collection tubes (Sarstedt AG, Nümbrecht, Germany) as practiced in the routine clinical settings. Ambulatory patients were asked to collect the feces at home and bring the feces during the next presentation in the department. Fecal sample from stationary patients have been collected in similar way. Prior to reaching the laboratory the samples were either stored at +4°C or at room temperature. Once the samples reached the laboratory, the samples were aliquoted and stored continuously at −30°C until further use.

### RNA isolation

Total RNA (including miRNAs) was extracted from stool specimens using Qiagen's miRNAeasy Mini Kits (Qiagen) as described previously [24]. Briefly, approximately 30–50 µl of frozen aliquoted stool was homogenized with RNase-free water to a total 150 µl and promptly lysed with 800 µl of QIAzol lysis reagent (Qiagen, Hilden, Germany). Precipitation was performed with chloroform and the aqueous phase was mixed with 1.5 volumes of 100% ethanol. Following elution, RNA quality was assessed using A260/A280 ratios using UV-spectroscopy and the samples were stored at −80°C until further analysis.

### MicroRNA quantification by real-time PCR

Quantitative miRNA expression analyses were performed using either TaqMan miRNA Assays (Applied Biosystems, CA, USA) or SYBRgreen method according to the manufacturers' instructions as described previously [24]. After reverse transcription, samples were run using iCycler IQ® detection system (Bio-Rad, CA, USA). To avoid an inter-plate bias/variability, miRNA expression analyses were performed using a single 96-well plate in duplicates. Differences between the groups are presented as ΔCt, which represent differences between the mean Ct value of the miRNA of interest and the mean Ct value of the normalizer miRNA. We used miR-16 for the fecal miRNA normalization in all samples as it has been shown to be most consistently expressed in different fecal samples including samples from colorectal cancer patients as described previously [24]. Selection of microRNAs was performed using following criteria: a) reported as pancreas-specific microRNA (miR-375) [25]; b) previously reported for potential implication in pancreatic carcinogenesis (miR-21, -196a, -210, -143, -155, -375, -216a) [26]–[28]; c) differential expression of miRNAs has been reported for blood (miR-21, -155, -196a, -210) [21]–[23] and/or pancreatic fluid (miR-21, -155; **Table 2**) [29]. Primer sequences for the RT-PCR assays are listed in the **Table S1**.

**Table 2:** The expression of analyzed fecal microRNAs in comparison to tumor tissues, pancreatic fluid and blood of patients with pancreatic cancer.

MicroRNA	Tumor	Pancreatic fluid	Blood	Feces	Predicted biological function	Gene Targets	References
**miR-143**	↑			↓	tumor suppression	KRAS	[37]–[38]
**miR-155**	↑		↑	↓	oncogene	TP53INP1	[34]
**miR-196a**	↑	↑	↑	↓	oncogene	ANXA1, KRT5, SPRR2C, S100A9	[40]–[41]
**miR-21**	↑	↑	↑	↔	oncogene	PTEN, RECK, PDCD4	[35]–[36]
**miR-210**	↑		↑	↔	oncogene	RAD52, FGFRL1	[39], [42]
**miR-216a**	↓			↓	tumor suppression	Ybx1	[45]
**miR-375**	↓			↔	tumor suppression	PDK1, JAK2, ATG7	[43]–[44], [46]
**References**	[25]–[28]	[29]	[21]–[23]				

### MicroRNA microarray expression profiling and data analysis

MicroRNA microarray expression profiling was performed with the aim to evaluate the presence of selected miRNAs in feces as previously described [24]. Briefly, total RNA was extracted from a single stool sample from a healthy subject using Qiagen's miRNAeasy Mini Kit as described above. Amplification and hybridization was performed according to the manufacturer's instructions (Illumina, Inc., San Diego, CA). Microarray data processing and analysis were performed using Illumina's BeadStudio software. Normalization has been performed using the Lumi Bioconductor software package creating log miRNA expression values [30]. Following conservative probe filtering step, which excluded probes that did not reach a detection value of P<0.05 the analyses resulted in the reliable detection for 630 probes.

### Statistical analysis

Data analyses were performed with GraphPad Prism 4.0 software (San Diego, CA, USA) or SPSS 17.0 (IBM Deutschland GmbH, Munich, Germany). The differences between two groups were analyzed using Student's t-test. The differences between more than two groups were analyzed using Kruskal-Wallis method with appropriate Dunn's multiple comparison *post hoc* test. Correlation analyses were performed using Pearson's test. Wherever appropriate, logarithmic regression was used to calculate the R^2^ and to create the equation of the slope. A two sided p-value of <0.05 was regarded as significant.

## Results

### Selection of fecal miRNAs

**Figure 1:**
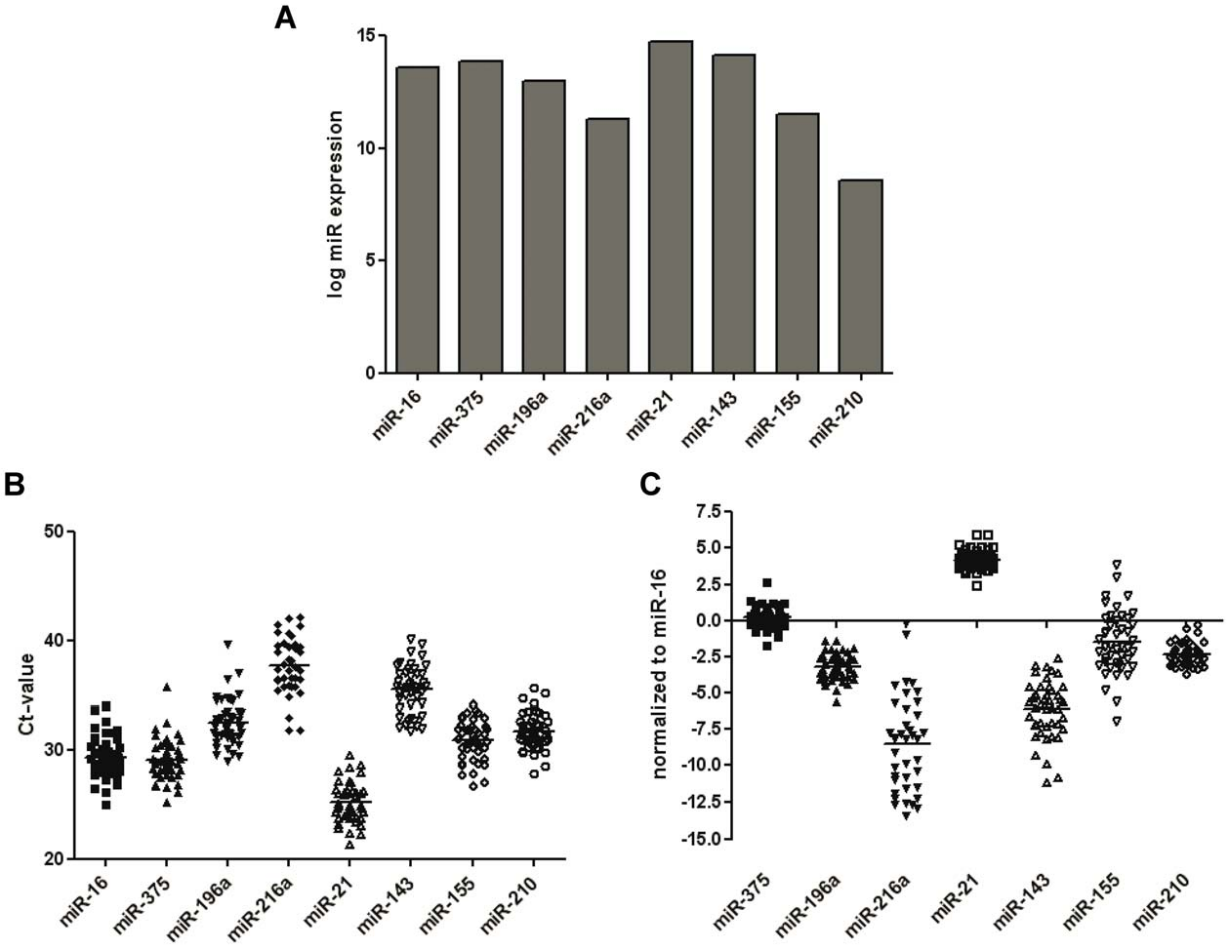
Detection of fecal miRNAs that are commonly dysregulated in pancreatic cancer tissues. (A) MiRNA microarray expression analyses were performed using Illumina microarray to evaluate the presence of selected miRNAs (miR-16, -375, -196a, -216a, -21, -143, -155 and -210) in a single stool sample of the healthy subject. Fecal miRNA expression was converted to log-expression values following Lumi Bioconductor normalization. (B & C) Expression of selected miRNA was confirmed in all 45 samples including controls, chronic pancreatitis and pancreatic cancer patients. Figure (B) represents the raw miRNA expression Ct-values for qualitative assessment. (C) Normalization was performed using standard ΔCt-method using miR-16 as internal fecal normalizer.

To investigate whether PCA patients have detectable fecal miRNA expression alterations, we first performed systematic analyses of the literature with the aim to select several miRNAs with highest evidence for miRNA deregulation in PCA patients. Based on the differential miRNA expression in pancreatic tumor tissues [18], [26]–[28], alterations in pancreatic juice [29] and in blood [21]–[23], as described above. We subsequently selected 5 upregulated (miR-21, -210, -143, -155, -196a) and 2 downregulated miRNAs (miR-216a and -375) for further analyses. **Table 2** provides a global overview of PCA-related miRNA expression alterations. To gain a global overview for the detectability of the selected miRNAs in feces in normal healthy subjects, fecal miRNA expression profiling was performed using miRNA microarrays using a single stool sample from the healthy volunteer. As shown in **Figure 1A**, all of the selected miRNAs were present in feces at detectable concentrations.

### Variation and normalization of fecal miRNAs

Next, we evaluated the miRNA expression in all samples simultaneously. Overall, all fecal miRNAs analyzed showed high degree of variation (over 10 Ct's) for the raw values (**Figure 1B**). To normalize the miRNA expression, ΔCt-values were calculated using miR-16 as an internal endogenous normalizer as described previously [24]. Following normalization, we discovered that the expression levels of miR-375, -21 and -210 demonstrated low (standard deviation (±SD) 0.76, 0.71, 0.78, respectively), and those of miR-216a, -143 and -155 (±SD: 3.31, 1.97, 2.15, respectively) showed high inter-individual variation across all samples (**Figure 1C**).

### Long-term stability of fecal miRNAs

**Figure 2:**
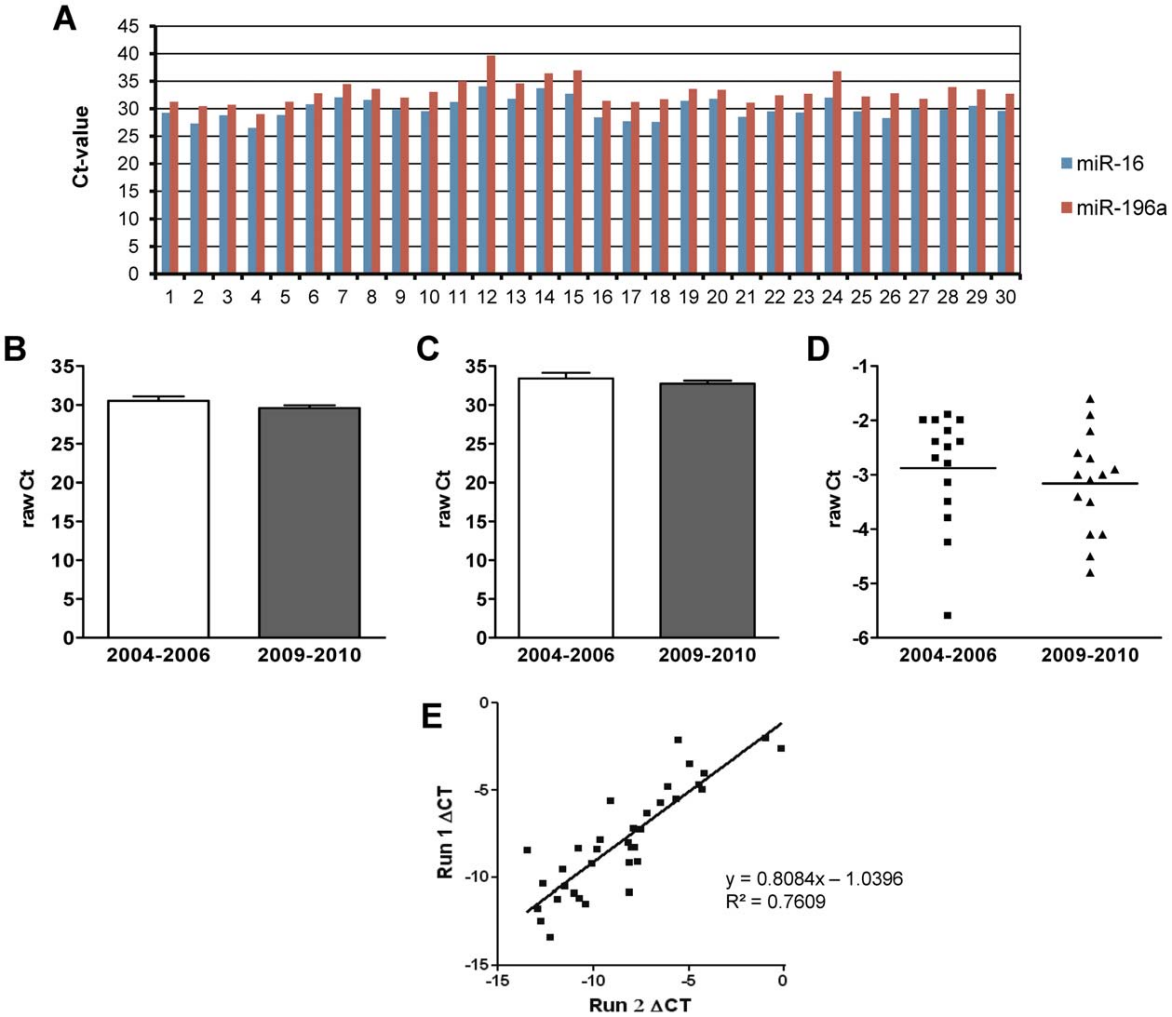
Stability of fecal miRNAs. To evaluate the long-term stability of the samples, we performed miRNA analyses in the fecal samples collected at different time points. Samples 1–15 were collected between 2004 and 2006 and samples 16–30 between 2009 and 2010 from healthy subjects. All samples were stored and processed in similar conditions. Figure (A) shows variations in miR-16 and miR-196a expression among all feces samples. (B) miR-16 and (C) miR-196a expression in subgroup analyses showed similar expression (p>0.1). (D) Normalized miR-196a expression is comparable in long- and short-term stored samples (p = 0.441). (E) Since miR-216a was present in feces at lowest concentrations, and its expression was analyzed by two independent quantitative RT-PCR runs to evaluate the reproducibility of the analysis (p<0.0001). Normalization was performed with using miR-16 as internal normalizer.

It has been previously shown that fecal miRNAs are stable in freshly collected samples or FOBT-kits [24], but it is not known whether miRNAs are stable during long-term storage. To test the stability of miRNAs under the long-term storage conditions, we analyzed fecal miRNA samples from an additional group of 30 healthy subjects. Among these, fifteen fecal samples (1–15) were collected between 2004 and 2006 and fifteen samples (16–30) between 2009 and 2010. As shown on the **Figure 2A**, all analyzed miRNAs demonstrated similar expression levels for both miR-16 and miR-196a. Mean±SD for miR-16 (**Figure 2B**) was 30.53±2.21 for samples after long-term storage compared to 29.59±1.39 for freshly collected samples (p = 0.172), and that of miR-196a was 33.41±2.84 vs. 32.75±1.43 (p = 0.426), respectively (**Figure 2C**). After normalization with miR-16, mean ΔCt-value for miR-196a was -2.88±1.03 for samples collected from 2004–2006 and −3.16±0.93 (p = 0.441) for samples collected during 2009–2010 (**Figure 2D**).

### Reproducibility of results for the miRNAs with low expression

Among all analyzed miRNAs, miR-216a was present in feces at lowest concentration (**Figure 1C**). To investigate if even miRNA with low expression can be reliably and reproducibly analyzed in feces, we performed two independent expression measurements. As demonstrated in **Figure 2E**, miR-216a expression was reproducibly detected in feces from all samples despite its low concentration (R^2^ = 0.761, p<0.0001).

### Differential expression of fecal miRNAs in PCA patients

**Figure 3:**
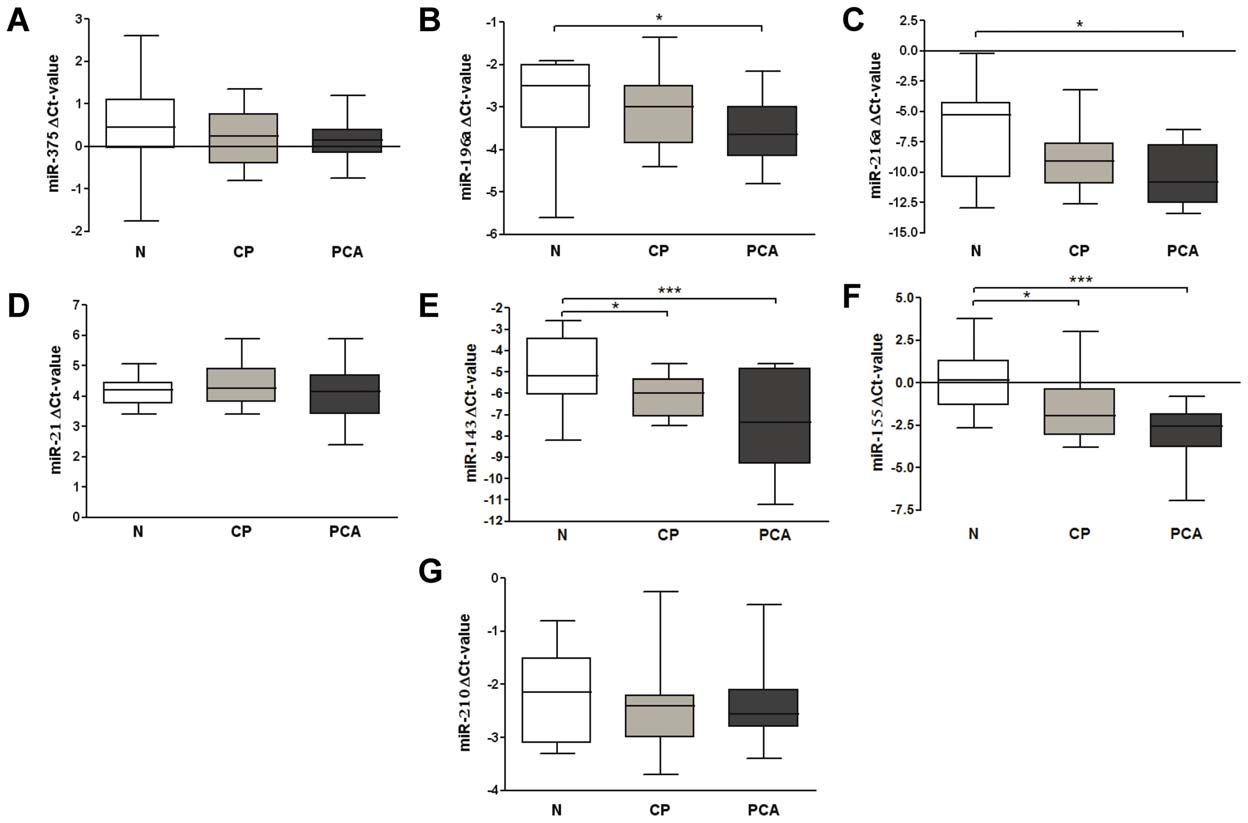
MiRNA expression patterns in patients with chronic pancreatitis and PCA. Figures (A) to (G) represent different miRNAs that were selected for the study based on the alterations in PCA tissues. *represents p<0.05, ***- p<0.001. Abbreviations: N-control subjects, CP- chronic pancreatitis, PCA- pancreatic cancer. The data are present as box-and-whiskers plots: the upper and lower limits of the boxes indicate the 75^th^ and 25^th^ percentiles, the lines inside the boxes - the medians, and the upper and lower horizontal bars denote the 90^th^ and 10^th^ percentiles, respectively.

To evaluate whether fecal miRNA expression analysis approach might be useful in PCA diagnosis, 45 fecal samples were evaluated. The clinical characteristics of the patients are shown in the **Table 1**. Fifteen fecal samples from controls were compared with fifteen each from patients with PCA and with chronic pancreatitis. All 7 tested miRNAs that are abundantly dysregulated in pancreatic cancer, were present in feces, although with specific variations in miRNA expression. Four of 7 miRNAs (miR-196a, -216a, -143 and -155) were expressed at significantly lower levels in stool samples from patients with PCA compared to controls (Kruskal-Wallis with Dunn's post test p<0.05 for miR-196a and miR-216a and p<0.001 for miR-143 and -155). Among these, only miR-143 and -155 reveal significant differences between patients with chronic pancreatitis and controls. Interestingly, subgroup analyses among the three groups revealed gradual decline in the *median* ΔCt for miRNA expression in fecal samples from controls to lower expression in patients with chronic pancreatitis and lowest in patients with PCA (**miR-196a**: −2.50 vs. −3.00 vs. −3.65; **miR-216a**: −5.30 vs. −9.10 vs. −10.80; **miR-143**: −5.20 vs. −6.00 vs. −7.35 and **miR-155**: 0.15 vs. −1.95 vs. −2.55 for controls vs. chronic pancreatitis vs. PCA, respectively) as shown in **Figure 3**. It is worth mentioning that in spite of the lower miRNA expression in feces from patients with PCA and chronic pancreatitis, miRNA expression differences were not statistically significant. Notably, miR-21, -375 and -210, which are frequently dysregulated in pancreatic cancers, were found to be unchanged in feces from controls and patients with PCA and chronic pancreatitis (**Figure 3**).

### A combination of miRNAs as diagnostic tool for PCA

**Figure 4:**
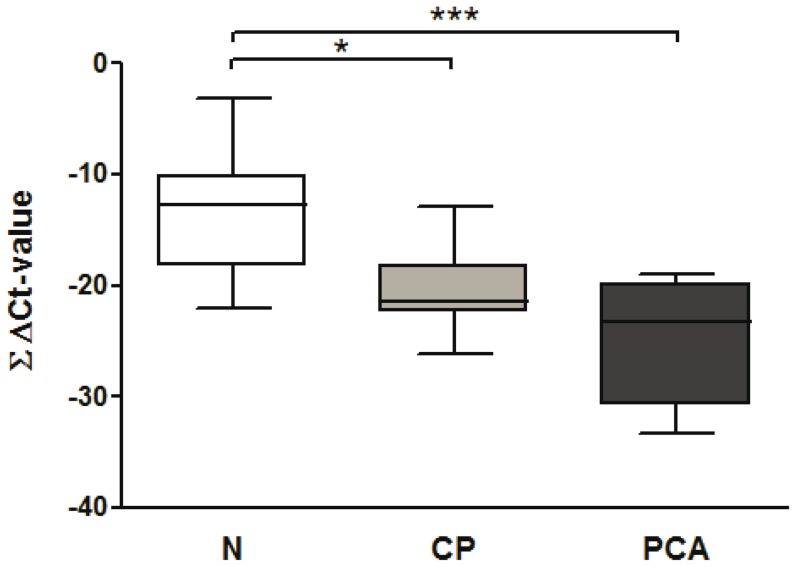
Cumulative miRNA expression analyses improve the separation of PCA samples. Summation of the ΔCt-values of miR-196a, -216a, -143 and -155 was performed to calculate the Σ ΔCt-value. *- p<0.05, ***- p<0.001. Abbreviations: N-control subjects, CP- chronic pancreatitis, PCA- pancreatic cancer. The data are present as box-and-whiskers plots: the upper and lower limits of the boxes indicate the 75^th^ and 25^th^ percentiles, the lines inside the boxes - the medians, and the upper and lower horizontal bars denote the 90^th^ and 10^th^ percentiles, respectively.

It was previously reported that expression pattern of several miRNAs rather than single miRNA would likely result in higher sensitivity and specificity for the identification of human cancers [28]. Based on this assumption we hypothesized that combination of 4 differentially expressed miRNAs would provide better discrimination of patients with PCA compared to controls. Since all 4 miRNAs showed gradual decrease in miRNA expression, we summarized ΔCt-values of miR-196a, -216a, -143 and -155 for each sample. As shown in **Figure 4**, the *median* ∑ΔCt-Values were: for controls −12.78 vs. −21.45 for chronic pancreatitis vs. −23.25 for PCA (Kruskal-Wallis test p<0.0002, Dunn's post test controls vs. chronic pancreatitis or PCA p<0.05 and 0.001, respectively). Using this combination, we achieved *median* ΔCt-Value difference of 10.47 (or 1418 fold) between PCA and controls, while ΔCt-value difference for miR-143 alone was 2.15 (or 4.4 fold).

## Discussion

In this proof-of-principle study, we evaluated feasibility of fecal miRNAs as potential biomarkers for the detection of PCA. Using a subset of miRNAs that are frequently dysregulated in PCA, we found that miR-196a, -216a, -143 and -155 are present at lower levels in fecal samples from patients with PCA compared to controls. In addition, we show that changes in fecal miRNA expression can also be detected in patients with chronic pancreatitis. Lastly, we demonstrate that a combination of a subset of miRNAs may provide a superior diagnostic value with a higher sensitivity and specificity for the identification of pancreatic neoplasia.

Apart from imaging techniques, there is a clear need for alternative modalities for the early detection of PCA. For example, the invasive collection of pancreatic juice or pancreatic duct brushing may seem potentially promising; this can only be performed in a limited number of patients due to its invasiveness and lack of suitability for screening purposes. Blood-based biomarker research has always been the focus, as it has tremendous potential for developing non-invasive screening approaches. However, feces have also been suggested as promising alternative, specifically for GI-related malignancies, due to constant shedding of exfoliated cells and production of pancreatic fluid [8], [24]. It is believed that fecal markers may even have higher diagnostic yield, since molecular cancer-related alterations in DNA, mRNA and miRNAs can be detected much earlier in feces. Recently, we and others have shown that detection of fecal miRNAs is a feasible approach that has a high degree of reproducibility, and that patients with colon neoplasia often show higher expression of miR-21 and -106a compared to controls [24], [31].

Currently, compelling evidence exists for tissue-specific alterations in miRNA expression in PCA [21], [26]–[28]. Those miRNAs have been the focus of multiple studies demonstrating complex interactions with key pathways and targets in pancreatic carcinogenesis. Based on existing knowledge for miRNA expression patterns in PCA, in the present study we selected 7 PCA-associated miRNAs that are either up- (miR-21, -155, -143, -210 and – 196a) or down-regulated (miR-375, -216a) for miRNA expression analyses in feces. In a pivotal study, Bloomston et al. demonstrated that PCA have distinct miRNA expression pattern with specific alterations of 25 miRNAs vs. benign pancreatic tissues and 21 miRNAs when comparing to chronic pancreatitis tissues. Using this miRNA expression pattern the authors could distinguish PCA tissues from benign and chronic pancreatitis tissues with accuracy of more than 90% [26]. Subsequently, it was shown that expression of miR-21 and -196a was associated with high proliferation (Ki67-index), presence of liver metastasis and prediction of poor survival/prognosis [19], [26]. Our data confirm previous reports demonstrating remarkable stability of fecal miRNAs in feces, as well as their high concentrations present in fecal samples [24], [32], [33]. Using target-based approach, we found differential miRNA expression of 4 out of 7 miRNAs (miR-196a, -216a, -143 and -155) in feces from patients with PCA in comparison to controls, while 3 miRNAs (miR-21, -375 and -210) were expressed at similar levels among various healthy subjects and patients. In this proof-of-principle study it was somewhat surprising to note that four of the differentially expressed miRNAs in our study showed decreased miRNA expression in feces of patients with PCA, while previous studies have demonstrated upregulation of miR-196a, -143 and -155 in pancreatic tumor tissues of patients with pancreatic cancer and down-regulation of miR-216a [26]–[28]. We hypothesized that the reason for the change in fecal vs. tissues expression of these miRNAs may be because of the alterations in pancreatic fluid outflow, which is frequently pathological in patients with pancreatic cancer and might be partially responsible for this discrepant observation. Because of the retrospective nature of the study, we were unable to gather data for the status of bile/pancreatic duct obstruction or perform paired tumor and fecal miRNA analyses. However, our hypothesis gains support from several another studies. First, it was previously shown that selected miRNAs (miR-21, -210, -155, -196a) were upregulated in plasma from patients with PCA, suggesting their mechanistic involvement in pancreatic cancers [21], [22]. Second, an independent study has demonstrated increased expression of miR-155 in pancreatic fluid of PCA patients [29], further supporting our observation. Third, our own observation for the gradual gradual decrease in the expression of selected miRNAs in feces of patients with chronic pancreatitis compared to controls and PCA patients support this argument. Nevertheless, we did not find any significant differences in fecal miRNA expression between PCA and patients with chronic pancreatitis that may reflect either the high risk for PCA development in patients with CP from one hand side or may potentially suggest a relationship to exocrine or endocrine pancreatic insufficiency.

In this study, miRNA profiling was solely used to gain global overview for the identification of miRNA expression patterns in fecal samples from pancreatic cancer patients. Since available samples were retrospectively collected and stored for long-term, we purposely avoided profiling of these tumor tissues in order to avoid potential selection bias using a high-throughput microarray-based technique. Even though selected miRNAs showed insignificant differences between CP and PCA patients, we believe that our results are thought provoking for PCA biomarker research, and provide a compelling springboard for undertaking additional prospective studies in the future to systematically evaluate potential differences in miRNAs that are unique to these patient subsets with regards to pancreatic and bile duct status. Results of our study highlight the need for further research on the biological significance of deregulated miRNAs in pancreatic neoplasia. As shown in the **Table 2**, most of the miRNAs analyzed in our study have multiple, cancer-related validated gene targets in human cancers. For example, Gironella et al. have shown that miR-155 targets Tumor protein 53-induced nuclear protein 1 (TP53INP1) disrupting its anti-tumoral activity in pancreatic cancer cells [34]. Several groups have shown that miR-21 targets PTEN, RECK, PDCD4, which may be responsible of oncogenic role of miR-21 [35], [36]. Likewise, miR-143, which is frequently deregulated in pancreatic cancers, has been shown to interact with KRAS [37] and restitution of tumor suppressor miRNA may inhibit tumor growth in xenograft pancreatic cancer model using nanovector delivery of miR-143/145 [38]. Additionally, miR-196a and miR-210 have been shown to play an oncogenic role by targeting several tumor suppressor genes (ANXA1, KRT5, RAD52) affecting proliferation and hypoxic stress [39]–[42], while miR-375 and -216a, which are frequently downregulated in pancreatic cancer, have been implicated in insulin secretion and in regulation of cell survival and proliferation by targeting several oncogenes such as PDK1, JAK2, ATG7 [25], [43]–[46]. This data are appealing for further intensive research of biological significance of miRNAs as biological effect of exosomal miRNA may be beyond the cellular or local tumoral effects [47], [48].

Current data implicate that miRNA expression patterns, rather than a single miRNA might be necessary for better utility for a miRNA-based diagnostic approach. For example, in our study, we performed a simple summation of the ΔCt values that allowed us to achieve a better discrimination of median ΔCt-values patients with PCA compared to controls. Such an approach may be of importance if fecal miRNA expression patterns are used for the screening of other GI malignancies. This is particularly relevant since fecal analysis is widely implemented in the clinical practice e.g. fecal occult blood tests for colorectal cancer screening and for the evaluation of pancreatic elastase in stool as a biomarker for exocrine pancreatic insufficiency. If validated in future, the vision of fecal miRNA-based approach for the identification of GI tumors could be easily translated into the diagnostics, an approach that will undoubtedly have a superior patient compliance compared to the currently available invasive diagnostic tests.

Although promising, fecal miRNA-based approach needs further intensive investigation prior to clinical application. As acknowledged previously, due to our ability to include only limited number of samples in this proof-of-principle study, the statistical significance of our results provides limited evidence for clinical applicability and potential of fecal miRNA for diagnosis of PCA patients in immediate future. Nonetheless, we believe that our findings are novel and future studies including prospective collection of the samples with various tumor stages with complete biochemical and clinical annotation for pancreatic/bile duct obstruction are needed because of potential impact of the pancreatic duct obstruction on fecal miRNA expression levels. Additionally, systematic analyses of feces using microarray or ultra-high throughput sequencing will help in uncovering novel fecal miRNAs and allow a methodological selection of potential biomarkers and their specificity for various diseases and conditions. Taking into consideration recent publication, where the slight increase of miR-16 has been described in KRAS^G12D^ transgenic animal model, high-throughput approaches will allow independent validation of specific microRNA as internal normalizers for PCA [49]. In light of these facts, ongoing research will have to elucidate the benefits of fecal miRNA not only in sporadic PCA but also in the high-risk populations that included patients with familial history.

In summary, we demonstrate that evaluation of fecal miRNAs can be effectively deployed in the PCA biomarker research. Differential expression of miRNAs in stool of patients with PCA suggests that fecal miRNAs can be attractive biomarkers for detection of PCA. Alterations in fecal miRNA expression levels offer the novel tool for further biomarker research for PCA screening in larger prospective studies.

## Supporting Information

Table S1 **Primers for microRNA RT-PCR.**
(DOC)
