# Supplementary material for: Feasibility of Fecal MicroRNAs as Novel Biomarkers for Pancreatic Cancer
Source: PLoS One. 2012 Aug 8;7(8):e42933. doi: 10.1371/journal.pone.0042933 (PMC3414456; doi:10.1371/journal.pone.0042933)
Supplement: Table S1 — Primers for microRNA RT-PCR. (DOC) [file pone.0042933.s001.doc]

**Table S1.** Primers for microRNA RT-PCR

| ***Name*** | ***TaqMan Assay ID*** | ***SyberGreen*** | ***miRBase Accession*** | ***References*** |
| --- | --- | --- | --- | --- |
| ***miR-143*** | 2249 |  | MIMAT0000435 |  |
| ***miR-155*** | 2623 |  | MIMAT0000646 |  |
| ***miR-16*** | 391 |  | MIMAT0000069 |  |
| ***miR-196a*** | 241070_mat |  | MIMAT0000226 |  |
| ***miR-216a*** | 2220 |  | MIMAT0000273 |  |
| ***miR-21*** |  | + | MIMAT0000076 | [24] |
| ***miR-210*** | 512 |  | MIMAT0000267 |  |
| ***miR-375*** | 564 |  | MIMAT0000728 |  |
